# Generation and Manipulation of Superoscillatory Hotspots Using Virtual Fourier Filtering and CTF Shaping

**DOI:** 10.1038/s41598-020-61674-z

**Published:** 2020-03-16

**Authors:** Abhijit Sanjeev, Nadav Shabairou, Arrad Attar, Daniel Scheberbaum, Yuval Kapellner, Moshe Sinvani, Zeev Zalevsky

**Affiliations:** 1 Faculty of Engineering and the Institute for Nanotechnology and Advanced Materials, Bar-Ilan University, Ramat-Gan, 5290002 Israel; 2 EKB Technologies Ltd, Bat-Yam, 59513 Israel; 3 Erlangen Graduate School in Advanced Optical Technologies (SAOT), Paul Gordan-Strasse 6, 91052 Erlangen, Germany

**Keywords:** Applied optics, Optical physics, Optical techniques

## Abstract

Superoscillation is a technique that is used to produce a spot of light (known as ‘hotspot’) which is smaller than the conventional diffraction limit of a lens and even smaller than the optical wavelength. Over the past few years, several techniques have been realized for the generation of the superoscillatory hotspot. In this article, for the first time to the best of our knowledge, we propose a novel and a more efficient technique for producing superoscillation in microscopic imaging by shaping the Coherent Transfer Function (*CTF*) of a lens via virtual Fourier filtering followed by a phase retrieval algorithm. We design and realize a phase mask which when placed at the pupil plane of a diffraction-limited lens produces a superoscillatory hotspot with sidelobes properly matched to the field of view (*FOV*) required in microscopic imaging applications, i.e. hotspot always coexists with huge intense rings known as ‘sidebands’ close to it and hence limiting the *FOV*. Our technique is also capable of extending the *FOV* with minimal loss in resolution of the hotspot generated and considerable ratio between the intensity of the hotspot to that of the side lobes while optimizing the obtainable *FOV* to the requirement of microscopy.

## Introduction

Imaging and microscopy have undoubtedly became an important tool in almost all fields of today’s technological world to see, visualize and analyze objects that are otherwise not seen by our naked eyes. Unfortunately, the lenses used in the optical microscope have a limit to the smallest resolvable feature. Before we talk about this limitation, it’s worth mentioning, how a lens forms an image of an object. An object scatters light at all angles due to diffraction. As the size of the object decreases the angle of diffracted light which carries the information about the object increases. So, while imaging smaller objects, conventional lens (due to limited aperture size) cannot collect all these light waves diffracted at a higher angle. Hence resulting in poor resolution of the image of the object. Ernst Abbe, in 1873, demonstrated that there is a limit to the resolution that a conventional lens can achieve and it is dependent on the wavelength of light, λ, and the numerical aperture (*NA*) of the objective lens to be: $d=0.61\lambda /NA$^1^. A microscope working in the air medium can have a max value of *NA* as 1. Hence the conventional diffraction limit is ~0.61*λ*. However, it is also possible to use immersion media to have *NA* greater than 1 because of its higher refractive index^2,3^.

Perhaps this fundamental limit has encouraged many researchers to come up with several techniques over the years to greatly enhance the resolution of optical microscope between tens and hundreds of nanometers. For instance, scanning near-field optical microscopy (SNOM) developed in 1984^4,5^, capture the evanescent near field waves close to the object. There have also been numerous other techniques utilizing the evanescent waves^6–10^. But these techniques are not suitable for thick samples like a biological cell because then it is required for the probe to be within tens of nanometers from the sample which limited its practicability. Other well-known superresolution techniques are: Structured illumination microscopy^11^ (SIM), Stimulated emission depletion (STED) by use of a doughnut beam^12^ and fluorescence localization microscopy^13^. The SIM techniques help in retrieving the lost higher spatial frequency content in an image by shifting the frequency content using spatially modulated light followed by a post-processing step to retrieve the high-resolution image. However, the improvement in resolution is only about twice that of the traditional microscopes. Additionally, the post-processing steps are complicated and time-consuming. Non-linear methods like STED and fluorescence localization microscopy, however, require either fluorescence labeling or post-processing to achieve superresolution. Recently, the concept of superoscillation has emerged significantly in the context of far-field, label-free and non-contact superresolution imaging.

Superoscillation is a mathematical concept in which a band-limited function is capable of having a local component that can oscillate faster than it’s fastest Fourier component^14,15^. Superoscillation in optics originated from the work of Torraldo di Francia in which he applied the concepts of super-directive antennas to an optical imaging system to see beyond the diffraction limit^16^. The idea of superoscillation was conceived and applied to many physical systems by Berry^17^. His works were mainly based on the work of Aharonov *et al*. on weak measurements in quantum systems^18^. Method for creation of optical superoscillation using a complex diffraction grating was proposed by Berry and Popescu^19^. Huang *et al*.^20^, demonstrated experimentally, for the first time, optical superoscillations by diffraction from a quasi-periodic array of nanoholes. Using superoscillatory techniques we can achieve a small spot which will be called from now as ‘hotspot’ which is less than the diffraction limit, while this is obtained at the expense of energy. In terms of optical superoscillations, a hotspot will always be accompanied by high-intensity side lobes which are higher in energy than the hotspot in the center.

In 2012, Rogers *et al*. developed a new superoscillatory microscope. They developed and fabricated a binary phase mask consisting of concentric rings that are based on binary particle swarm algorithm^21^. Creation of super-oscillating (SO) beams with sub-diffraction limited features have been developed by modulating the lens’ pupil^21,22^ and by superposition of Bessel beams^23–25^. A super oscillatory function could also be constructed using a complete set of prolate spheroidal wavefunctions (PSWFs)^26^. The concept of design of annular pupil phase filters for the generation of superoscillation was proposed by Cagigal *et al*. in 2004^27^. In 2017 Singh *et al*. developed a method using Spatial Light Modulator (SLM) for shaping the sub-diffraction hotspot obtained and using it to trap a single nano-particle^28^. In 2018, Rogers *et al*. with the use of PSWFs achieved simultaneously optimization of spot size and Field of view^29^. In 2019, Shapira *et al*. investigated methods to produce multi-lobe optical superoscillating beams, with nearly constant intensity and constant local frequency and successfully tested in structured illumination microscopy^30^.

Though the techniques described above are undoubtedly powerful in producing superoscillatory hotspots, most of them use strong mathematical calculations for the formulation of the phase mask that is needed for the creation of the hotspot or they use some sort of optimization algorithm to attain it. In our research, we propose a novel technique in which we design a phase mask which when applied to a lens can produce a superoscillatory spot. The main advantage of our technique is that we use a simple Coherent Transfer Function (*CTF*) shaping computed digitally in a computer by using virtual Fourier filtering without the need of using any complex mathematical functions or calculations. We also show that we are capable of tailoring different hotspot size with varying FOV. Specifically, those that match well the microscopy applications in which imaging of cells having a FOV being around 10–15 microns. We show through simulation, that indeed it is possible to go beyond the classical diffraction limit using our technique. As proof of our concept, we experimentally apply our technique on a digital lens that is being realized with a spatial light modulator (*SLM*) having $NA=0.0075\,at\,\lambda =543\,nm$.

## Materials and Methods

### Principle

**Figure 1:**
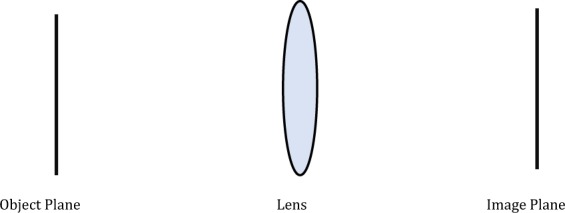
Typical coherent imaging system.

Let us consider a coherent imaging system as shown in Figure 1. This can be described as a convolution operation between the coherent impulse response of the system and the field at the object plane described by Eq. 1

1 $${U}_{i}=h(x,y)\otimes {U}_{o}$$

where ${U}_{o}$ and ${U}_{i}$ are the field at object and image plane respectively, *h* is the coherent impulse response of the imaging system, $\otimes$ is the convolution operator and *x*, *y* are the coordinates of the image plane. Let *H* be the Fourier transform of *h*. The impulse response *h* in our case is the point spread function of the lens (*psf*). In the frequency domain the corresponding spectra of Eq. 1 can be written as

2 $${G}_{i}(u,v)=H(u,v)\,{G}_{o}(u,v)$$

where H is the coherent image transfer function (*CTF*), *u*, *v* are the frequency coordinates. H can be described as:

3 $$H(u,v)=P(\,-\,\lambda {z}_{f}u,-\lambda {z}_{f}v)$$

where *P* is the pupil of the system, *λ* wavelength of the light used and *z*_*f*_ the focal length of the imaging system. For a lens, the pupil function is a circular aperture given by

4 $$P={\rm{circ}}(\sqrt{({x}^{2}+{y}^{2})/w)})$$

where *w* is the radius of the aperture of the lens. From Eqs. 3 and 4 we have an expression for H as:

5 $$H={\rm{circ}}(\sqrt{({u}^{2}+{v}^{2})/{f}_{0})})$$

where *f*_0_ is the cut off frequency of the CTF given by:

6 $${f}_{0}=\frac{w}{\lambda \,{z}_{f}}$$

where *z*_*f*_ is the focal length of the lens.

**Figure 2:**
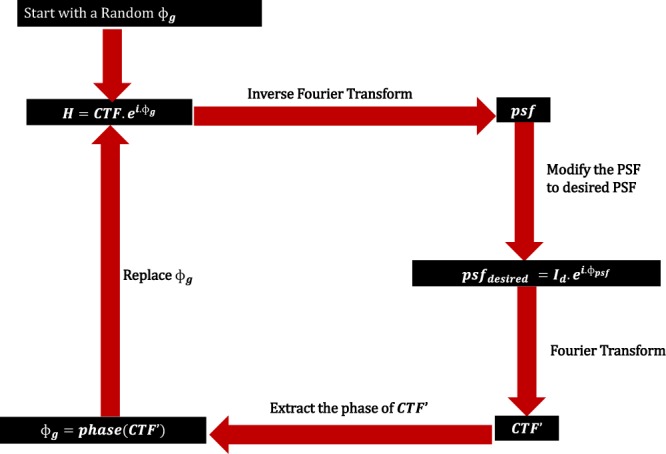
Steps involved in regular CTF shaping.

Phase retrieval techniques like the Gerchberg-Saxton (GS) algorithm deal with the problem of obtaining the phase of a light field by just knowing the modulus of its Fourier transform^31^. Hence using this technique, it is possible to engineer the *psf* of a lens^32–34^. In our technique, we use a modified GS algorithm to engineer the *psf* of the lens to obtain a hotspot. Before we discuss the modification, it’s worth mentioning the existing GS algorithm for *psf* shaping. The basic idea of *psf* shaping is to apply a phase mask in the lens pupil plane, such that the *psf* of the lens is shaped to the desired intensity profile. The advantage of this technique is that it is done digitally on a computer. Figure 2 sketches the steps involved in the G.S algorithm to obtain the phase mask that can be used to shape the *psf*.

The algorithm starts with an assumption of a random phase mask with phase ${\Phi }_{{\rm{g}}}$, varying from 0 to 2π phase. This phase mask is multiplied with the pupil function of the lens, i.e with the *CTF* to obtain a new *CTF* denoted as

7 $${H}_{new}=CTF.{e}^{i.{\Phi }_{{\rm{g}}}}$$

We then proceed to take the inverse Fourier transform of *H*_*new*_, which would yield the *psf* of the system corresponding to *H*_*new*_ in the *psf* plane. In the following step, we preserve the phase of the *psf* and set the amplitude as our desired amplitude pattern. We then perform Fourier transform on the $ps{f}_{desired}$ to yield $CTF{\prime}$ in the *CTF* plane. In the next step value of ${\Phi }_{{\rm{g}}}$ is updated by extracting the phase from $CTF{\prime}$. This is one iteration. This is continued till there is a convergence of the *psf* to the desired *psf*. The result is a phase mask with a phase ${\Phi }_{{\rm{g}}}$ which when placed at the pupil plane of the lens will result in a shaped *psf*. This is a very powerful technique to shape the *psf*. The requirement is to know the desired amplitude pattern beforehand. It is possible to construct a hotspot amplitude pattern mathematically and set it as desired *psf* amplitude to obtain the phase mask. However, the mathematical construction of such hotspots already yields the corresponding phase masks that produce such spots. Hence, there is no need to do such an iterative algorithm for obtaining the same. Also, mathematically it is hard to construct such hotspots.

**Figure 3:**
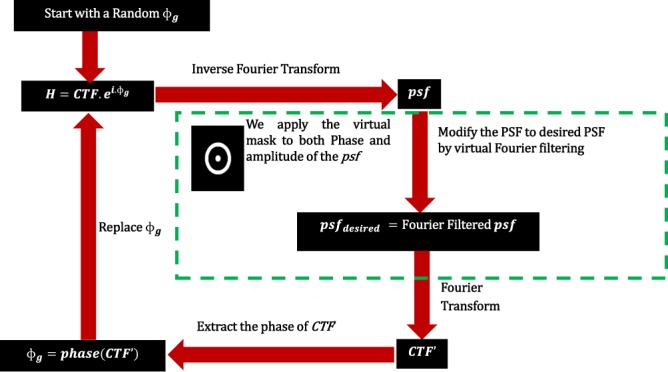
Steps involved in the modified Algorithm for CTF shaping.

Using our novel technique, we show that indeed without the prior knowledge of such mathematically formulated hotspot amplitudes, it is possible to create a phase mask that can produce hotspots with a small modification to the existing GS algorithm. Figure 3 explains the modified G.S Algorithm for shaping the *psf* to a superoscillatory hotspot.

**Figure 4:**
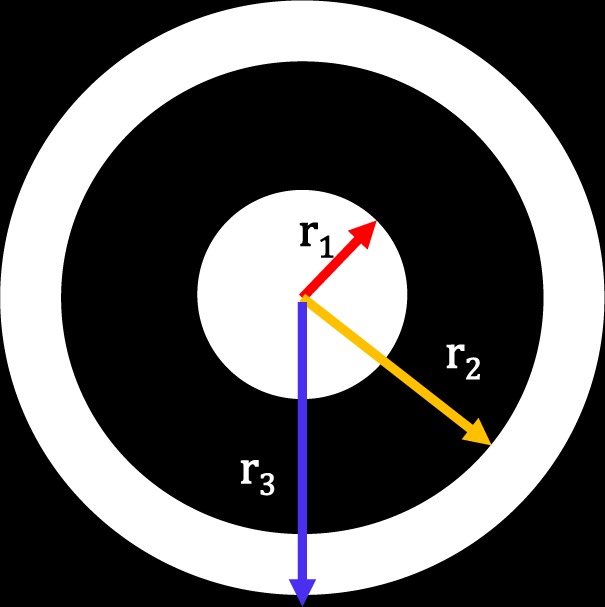
Virtual Fourier Filter Mask.

The modification is shown in the green dotted box, where instead of setting the desired amplitude pattern to the desired *psf*, we perform a virtual Fourier filtering of the *psf* using a filter mask. The filter mask has a circular region with a radius *r*_1_, and an annular region with an inner radius *r*_2_ and outer radius *r*_3_. The criteria for the selection of these radii will be discussed later in the simulation section. The mask takes the following functional value: (please refer to Figure 4)

8 $$Mask=\{\begin{array}{ll}1, & 0\le r\le {r}_{1}\,and\,{r}_{3}\ge r\ge {r}_{2}\\ 0, & else\end{array}$$

In the modified step, we multiply the virtual mask to the extracted phase of the *psf* (denoted by *θ*)and set amplitude as that of the mask (*m*). Hence the filtered *psf* can be written as

9 $$ps{f}_{desired}=m.{e}^{i.m.\theta }$$

The remaining steps are the same. After several iterations, the *psf* will converge to a hotspot depending on the right choice of the filter mask. In the following section, we show how it can be done through MATLAB simulation.

### Simulation

In the simulation, we show results for 3 different wavelengths ($\lambda =532\,nm,460\,nm$ and 632 *nm*) for a lens with the same *NA* (equals 1). The initial step in the simulation is to set the pixel size of the *psf* plane $(dx=6.65\,nm)$. The physical length, *L* of the *psf* plane is given by

10 $$L=M\,dx$$

where *M* is the number of pixels in the x-direction. We have set *M* = 1024 pixels. Now we can formulate the *CTF* plane based on the *psf* plane. They both are related by a Fourier transform. Hence the pixel size of the *CTF* plane is given by

11 $$df=\frac{1}{L}$$

**Figure 5:**
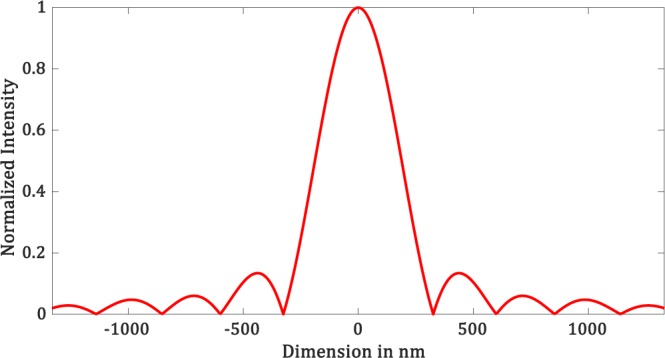
Amplitude plot of PSF of the simulated lens.

We then define the lens aperture radius *w* and the focal length *z*_*f*_. Based on Eq. 6, we can calculate the cut off frequency in the $CTF$ plane. Hence, we can define the *CTF* of the lens based on Eq. 5. It is a circle of radius *f*_0_. The inverse Fourier transform of *CTF* yields the diffraction-limited *psf*. Figure 5 shows the amplitude plot of the diffraction-limited *psf* for ($\lambda =532\,nm,\,NA=1,\,dx\,=6.65\,nm$).

The radius of the Airy disk is given by $(1.22\lambda {z}_{f}/D)$, where D is the aperture of the lens. Hence the total width of the airy disk is given by $(d=1.22\lambda /NA)$. In the case of $\lambda =532\,nm$, *NA* = 1, we have a diffraction-limited spot with $d \sim 649$ nm.

Now we can move forward towards designing the virtual filter mask to be applied on the *psf*. The mask is a binary filter mask as described by Eq. 8. We have three parameters *r*_1_, *r*_2_ and *r*_3_. Value of *r*_2_ and *r*_3_ can be set at the beginning. The choice of *r*_2_ and *r*_3_ is made based on the amplitude plot shown in Figure 5. The value of *r*_2_ and *r*_3_ is the radius where the *psf* amplitude first goes to zeros and then increases to maximum respectively. For $\lambda =532\,nm$, we have *r*_2_ = 325.8 *nm* and *r*_3_ = 438.9 *nm*. Once we set these two parameters, the selection of *r*_1_ is crucial in the formation of hotspots. *r*_1_ can take any values between 0 to 325.8 *nm*. Pixel wise there are 49 values for *r*_1_. We tried several masks with all possible values of *r*_1_ and came to the following conclusions. We found that there is an upper and lower limit of *r*_1_ that would help us achieve the hotspot after *CTF* shaping. Value of *r*_1_ should be such that the ratio between *r*_1_ and *r*_2_ should be greater than 0.25. This sets the lower limit for *r*_1_. The upper limit for *r*_1_ is such that it *r*_1_ should be less than the *FWHM* of the diffraction-limited amplitude plot. Once we design the mask, for each mask we perform the steps mentioned in Figure 3.

**Figure 6:**
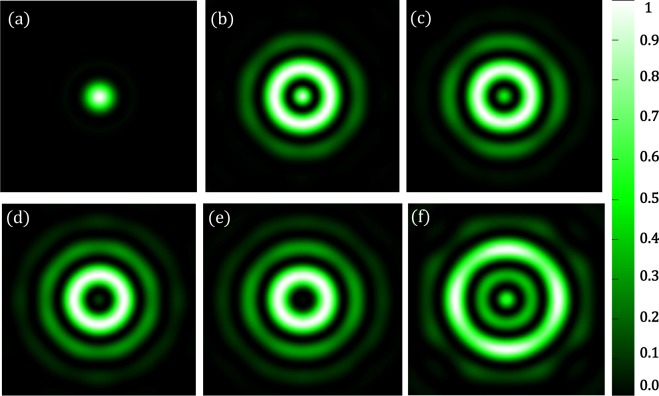
Simulation results for $\lambda =532\,nm,\,NA=1$ (**a**) Diffraction-limited spot of size $324.5\pm 6.65\,nm$. Superoscillatory hotspot of sizes (**b**) $179.2\pm 6.65\,nm$ (**c**) 153.1 ± 6.65 *nm* (**d**) 113.2 ± 6.65 *nm*. (**e**) 86.6 ± 6.65 *nm* (**f**) Superoscillatory hotspot of size 166.4 ± 6.65 *nm* with an extended FOV of 1123.7 ± 6.65 *nm*.

**Figure 7:**
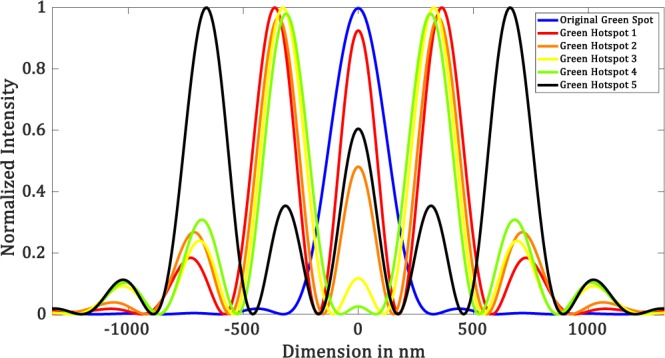
Intensity profile plots of the simulated results in Figure 6. Blue plot refers to refers to the intensity profile plot of Figure 6(a), red plot refers to the intensity profile plot of Figure 6(b), black plot refers to the intensity profile plot of Figure 6(f), orange plot refers to the intensity profile plot of Figure 6(c), yellow plot refers to the intensity profile plot of Figure 6(d) and green plot refers to the intensity profile plot of Figure 6(e).

**Figure 8:**
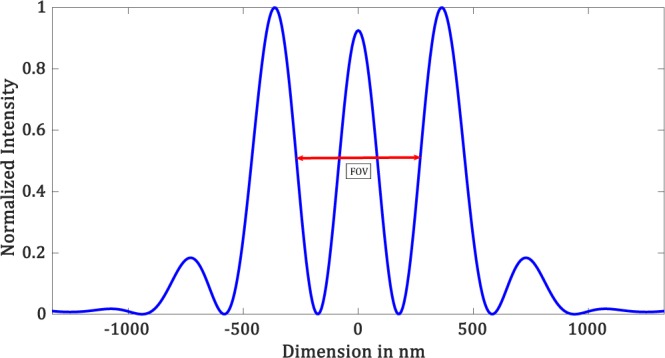
FOV measurement.

Each mask takes about 100 iterations to yield a hotspot. In Fig. 6(a–e), we show the diffraction-limited spot as well as a few such hotspots created using our simulation technique for $\lambda =532\,nm$. Figure 7 shows the corresponding intensity profiles. The parameters of the masks are shown in Table 1. We have seen that, for the creation of hotspot *r*_1_ takes values such that $\,86.45\le {r}_{1} < 192.85$. It is interesting to note that the region between *r*_1_ and *r*_2_, where the mask is zeros. This region is related to the field of view (*FOV*) of the superoscillatory spot. *FOV* is measured between the two points on the side lobes as shown in Figure 8. It forces the algorithm to set the phase and intensity of the *psf* in this region to be zero. So larger the zero region between *r*_1_ and *r*_2_ is, the smaller will be the hotspot being produced. Hence, we have a tenability with regard to the size of the hotspot obtained. Also, by forcing the field at those points to be zero, the intensity is redistributed such that it forms a superoscillatory spot.

**Table 1:** Fourier filter mask’s parameters for $\lambda =532\,nm,\,NA=1$.

MASK #	*r*_1_ (*nm*)	*r*_2_ (*nm*)	*r*_3_ (*nm*)
1	86.45	325.8	438.9
2	133	325.8	438.9
3	166.25	325.8	438.9
4	186.2	325.8	438.9
5	79.8	598.5	718.2

As previously mentioned, the hotspots are always accompanied by huge intense sidelobes which limit the *FOV* and hence its applicability. Using the proposed technique, we could also increase the *FOV* by designing another filter mask such that we still obtain a hotspot with sufficiently good resolution below the diffraction limit and at the same time have an increased *FOV*.

This is because our filter design is such that between the hotspot and the main side lobe there is a minor side lobe whose peak intensity is smaller than the hotspot. We will now describe how to choose the value of the radii of such a mask that would produce a hotspot with increased FOV.

Referring to Figure 5, we will set the value of *r*_2_ and *r*_3_ based on the second minor sidelobes. Value of *r*_2_ = 598.5 *nm*, where the amplitude drops to zero and *r*_3_ = 718.2 *nm*, where the amplitude then goes to a maximum. Now choosing of *r*_1_ will yield us a hotspot. We found that a hotspot is obtained when the value of *r*_1_ is such that the ratio between *r*_1_ and the point where the amplitude of the *psf* first drops to zeros (i.e. 325.8 *nm*) is less than 0.25, which means it can take values between $0 < {r}_{1}\le 79.8$. But not all values yield increased *FOV*. The upper limit value will yield a hotspot with increased *FOV*. As the value of *r*_1_ is decreased from the upper limit we see that there would be an increase in the intensity of the minor sideband with respect to the hotspot which will destroy the *FOV* of the hotspot. We simulated the increased *FOV* for the case in which *r*_1_ is set to 79.8 *nm* and yields an increased *FOV*. Figure 6(f) shows the hotspot with increased *FOV* of 1123.7 ± 6.65 nm with a hotspot size of 166.4 ± 6.65 nm very well below the diffraction limit. Now we can have an analysis of the intensity profile plots in Figure 7. Blue plot refers to the diffraction-limited spot size of $324\,\pm \,6.65\,nm$. Table 2 consolidates the obtained hotspot sizes and the corresponding FOVs. We obtain a range of hotspot sizes from 86.6–179.2 nm. There is an increase in *FOV* from 498.7 ± 6.65 nm to 1123.7 ± 6.65 while keeping the increase in hotspot size considerably less from 153.1 nm to 166.4 nm. (Refer Figure 6(c,f)).

**Table 2:** Simulation Result Analysis with respect to Hotspot size and *FOV* to the mask used for $\lambda =532\,nm,\,NA=1$.

Mask #	Figure #	Hotspot FWHM (*nm*)	FOV (*nm*)
_1_	6 (b)	179.2 ± 6.65	738.5 ± 6.65
2	6 (c)	153.1 ± 6.65	498.7 ± 6.65
3	6 (d)	113.2 ± 6.65	458.5 ± 6.65
4	6 (d)	86.6 ± 6.65	432.1 ± 6.65
5	6 (f)	166.4 ± 6.65	1123.7 ± 6.65

**Figure 9:**
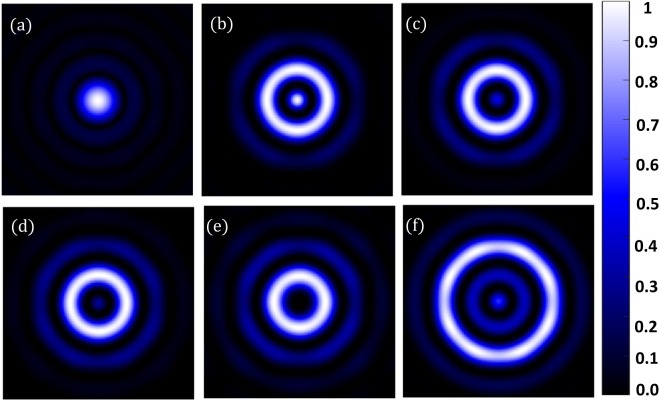
Simulation results for $\lambda =460\,nm,\,NA=1$ (**a**) Diffraction-limited spot of size $280.6\pm 6\,nm$. Superoscillatory hotspot of sizes (**b**) $166.1\pm 6.65\,nm$ (**c**) $139.5\pm 6.65\,nm$ (**d**) 112.9 ± 6.65 *nm*. (**e**) $46.4\pm 6.65\,nm$ (**f**) Superoscillatory hotspot of size $126.2\pm 6.65\,nm$ with an extended FOV of $964.1\pm 6.65nm$.

**Figure 10:**
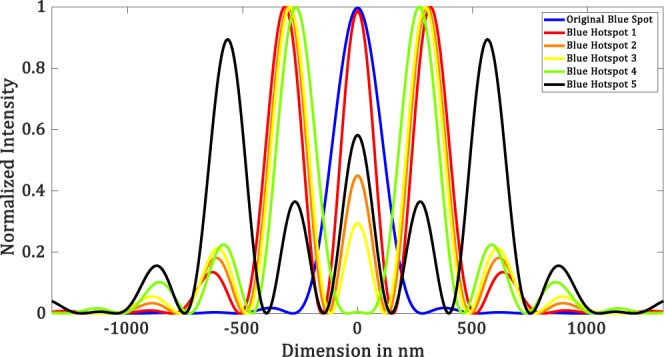
Intensity profile plots of the simulated results in Figure 9. Blue plot refers to the intensity profile plot of Figure 9(a), red plot refers to the intensity profile plot of Figure 9(b), black plot refers to the intensity profile plot of Figure 9(f), orange plot refers to the intensity profile plot of Figure 9(c), yellow plot refers to the intensity profile plot of Figure 9(d) and green plot refers to the intensity profile plot of Figure 9(e).

**Figure 11:**
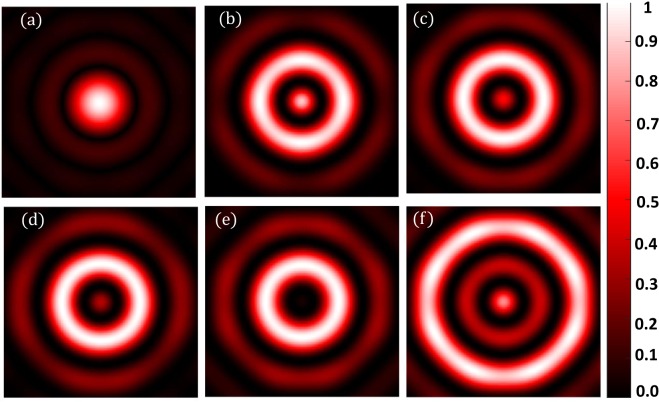
Simulation results for $\lambda =632\,nm,\,NA=1$. (**a**) Diffraction-limited spot of size $3\pm 6\,nm$. Superoscillatory hotspot of sizes (**b**) $192.7\pm 6.65\,nm$ (**c**) $179.7\pm 6.65\,nm$ (**d**) $166.5\pm 6.65\,nm$. (**e**) $113.2\pm 6.65\,nm$ (**f**) Superoscillatory hotspot of size $206.3\pm 6.65\,nm$ with an extended FOV of $1336\pm 6.65\,nm$.

**Figure 12:**
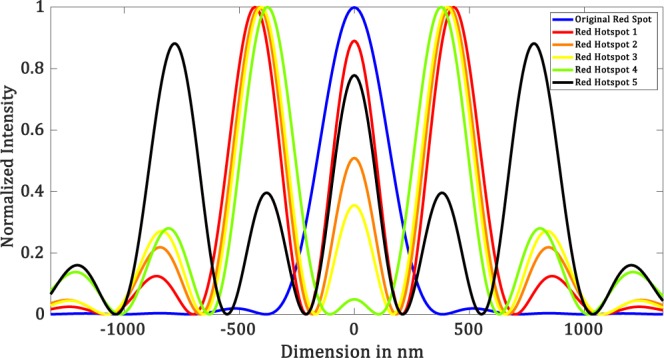
Intensity profile plots of the simulated results in Figure 11. Blue plot refers to the intensity profile plot of Figure 11(a), red plot refers to the intensity profile plot of Figure 11(b), the black plot refers to the intensity profile plot of Figure 11(f), the orange plot refers to the intensity profile plot of Figure 11(c), the yellow plot refers to the intensity profile plot of Figure 11(d) and green plot refers to the intensity profile plot of Figure 11(e).

Simulation is done for other wavelengths as well ($\lambda =460\,nm$ and 632 *nm*). We have kept the *NA* to be the same as the previous one. Figures 9–12 shows the results corresponding to these wavelengths. The mask parameters corresponding to each wavelength are tabulated in the supplementary material (Tables S1 and S2). In the supplementary material, we also show that our method can be generalized to any wavelength and *NA*. And we have seen that the criteria of selection of the mask parameters remain unchanged with wavelength and *NA*.

Using our simulation (for $\lambda =532\,nm$), we obtained a phase mask with 24 × 24 pixels with a pixel size that corresponds to the *CTF* plane given by:

12 $$\delta =\lambda .{z}_{f}.df$$

where $\lambda ,{z}_{f},df$ is the wavelength of the light, focal length of the lens and pixel size of the *CTF* plane respectively. In our case $\delta =335\,\mu m$. But since the physical dimension of the lens that we chose is $8.3\,mm$, which is equal to the pupil size, we will need to re-size the obtained phase mask to match the physical size of the pupil of the lens. In real experiments, it can be realized by applying the synthesized mask on the lens aperture using an SLM. The 24 × 24 pixels mask can be resized according to the pixel size of the SLM. The typical pixel size of an SLM is ~8 µ. For instance, by merging 45 pixels together we can convert 24 × 24 pixels to 1080 × 1080 pixels which is the typical resolution of our SLM. This is done to reduce the computational load. Else we would need to compute the phase mask for 1080 × 1080 pixels of the SLM which can be done only by zero-padding the 1080 × 1080 pixels sufficiently to a higher number, which eventually will increase the computational load.

## Results and Discussion

**Figure 13:**
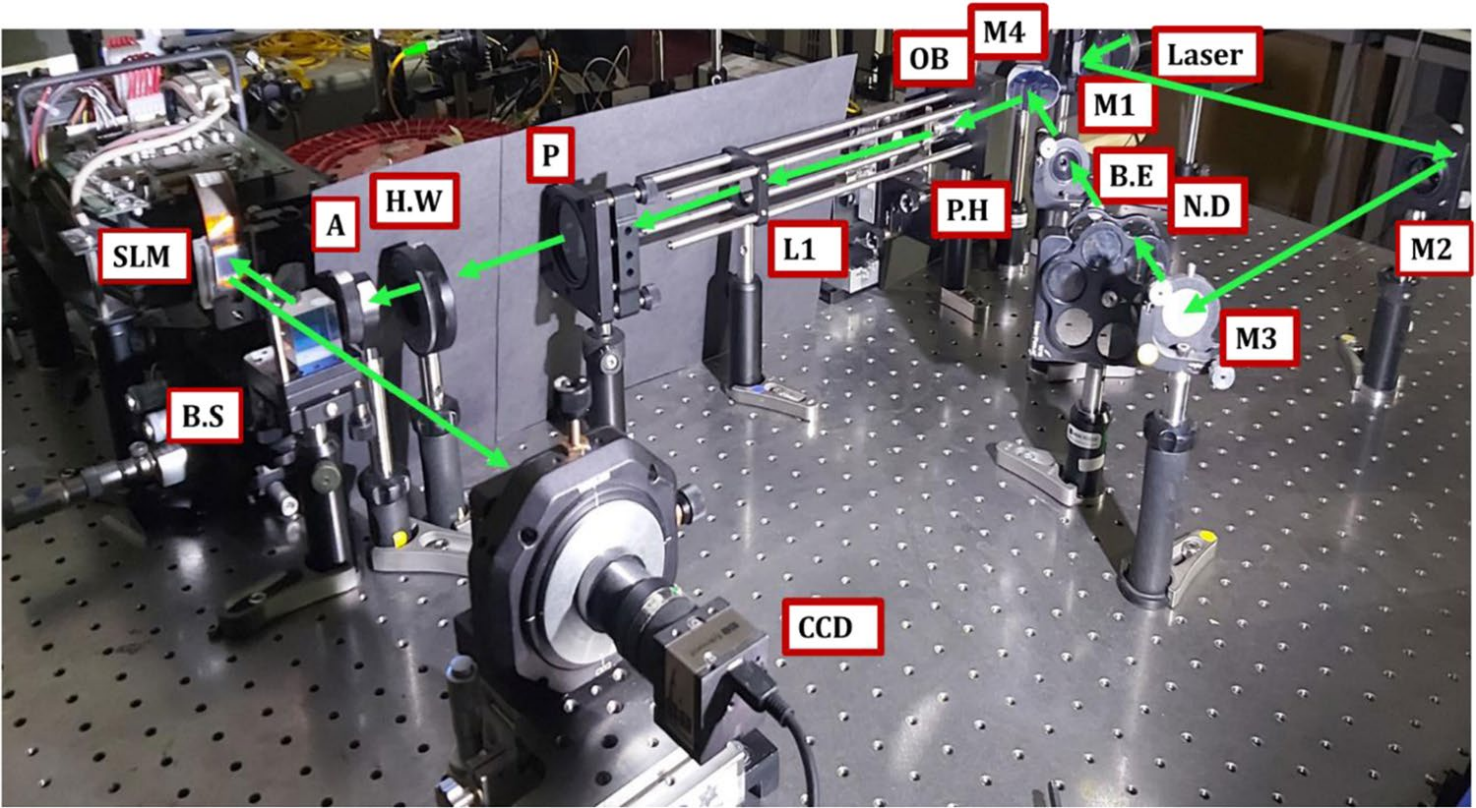
Experimental setup for producing super oscillatory hotspots. 543 nm laser passes through a variable Neutral Density filter N.D reflected from mirrors M1, M2, and M3. It is then expanded using a beam expander B.E. and is reflected using mirror M4 to the back aperture of a microscope objective OB (25X). It is then pinhole filtered by P.H of size 15 µ and collimated using lens L1 of focal length 200 mm. Polarizer P polarizes the beam, halfwave plate H.W sets the polarization to the working polarization of the phase-only SLM. Aperture A sets the size of the illuminating beam on SLM. Beam splitter B.S reflects the beam to illuminate the SLM and transmits the phase-modulated light towards the CCD.

**Figure 14:**
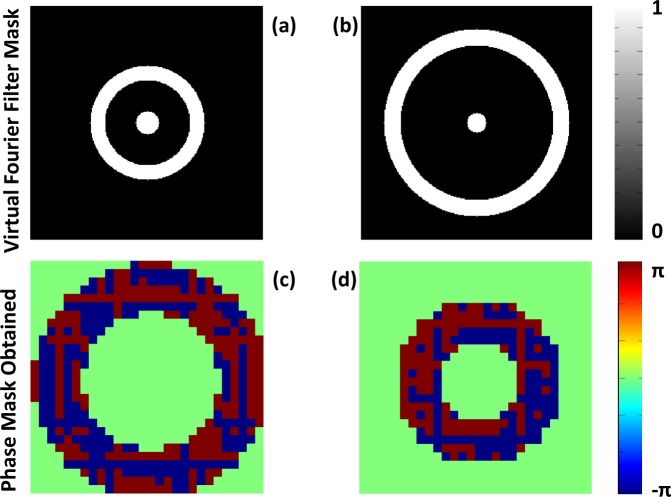
(**a**) Virtual Fourier filter mask 1. (**b**) Virtual Fourier filter mask 2. (**c**,**d**) The obtained phase mask corresponding to mask 1 and mask 2 respectively after the CTF shaping. The phase mask is resized to fit the SLM pixel size of 8 µm.

In order to show the proof of concept experimentally, we programmed a lens phase function in a computer-generated hologram and displayed it on our Spatial Light Modulator (SLM, HoloEye, 1080 × 1920 pixels, 8 *μm* pixel size) such that it produces a diffraction-limited spot corresponding to a lens of $400\,mm$ focal length at a distance of 400 *mm* from the SLM in +1 or −1 diffraction order. Figure 13 shows the experimental setup we used. We used a spatially filtered collimated and well-expanded laser beam (543 nm Coherent Laser) to illuminate the SLM. We illuminated only a circular region of the total pixels with a diameter of 750 pixels. Hence the lens function on the SLM is equivalent to a lens of NA = 0.0075. The theoretical FWHM of the Airy disk diameter of such a lens is given by $(0.61\lambda /\,NA)=44.16\,\mu m)$. The focal spot was captured directly on a CCD sensor (Edmund Optics Camera 1920 × 2560 pixels) with a pixel size of 2.2 *μm*. Figure 14(a) shows the diffraction-limited hotspot obtained when we applied the hologram that corresponds to the lens function on the SLM. The spot size is about 57.6 ± 2.2 *μm*. SLM plane deviations and noise resulted in a larger spot than expected. Despite these aberrations, we were able to obtain the hotspot and its dimension is close to the one designed in simulations.

The simulation was performed on a lens that is equivalent to an *NA* of 0.0075. We have already discussed the criteria for the selection of the virtual mask that we use to perform the Fourier filtering. We have used 1024 × 1024 pixels in simulation with a pixel size of the *psf* plane as 1 *μm*. We have used two masks for CTF shaping, mask 1 and mask 2 (Figure 14(a,b)) that yield a phase mask that produces superoscillatory hotspot of *FWHM* = 23 ± 1 *μm* and 25 ± 1 *μm* respectively. Figure 14(c,d) show the phase mask obtained after *CTF* shaping. The obtained phase mask is encoded in a computer-generated off-axis hologram and is applied on the SLM which already has a hologram with the lens function. So, it will be equivalent to the case in which we are placing a phase mask in the pupil plane of the lens. Mask 1 has parameters of *r*_1_ = 12 *μm*, *r*_2_ = 44 *μm* and *r*_3_ = 59 *μm*. Mask 2 has parameters of *r*_1_ = 10 *μm*, *r*_2_ = 80 *μm* and *r*_3_ = 97 *μm*.

**Figure 15:**
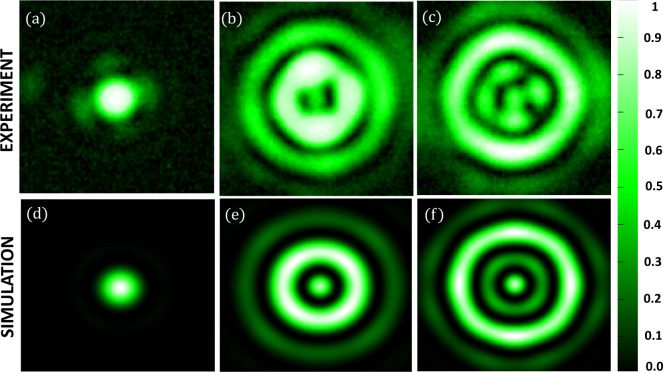
(**a**–**c**) Show experimental data and (**d**–**f**) show simulated data. (**a**) Diffraction limited spot of the size of ∼57.36 ± 2.2 μ*m*. (**b**) Hotspot of size ∼25.6 ± 0.2 μ*m*. (**c**) Hotspot of size ∼26.6 ± 0.2 μ*m*. (**d**) Simulated Diffraction-limited spot of size 27.5 ± 1 μ*m*. (**e**) Hotspot of size 25 ± 1 μ*m* (**f**) Hotspot of size 25 ± 1 μ*m* with increased FOV.

**Figure 16:**
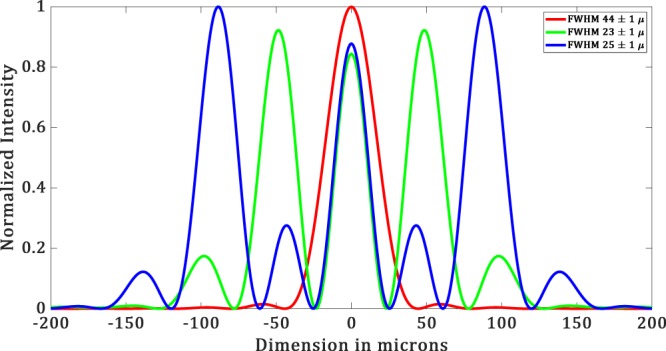
Intensity profile plots of the simulated images in Fig. 15(d–f).

**Figure 17:**
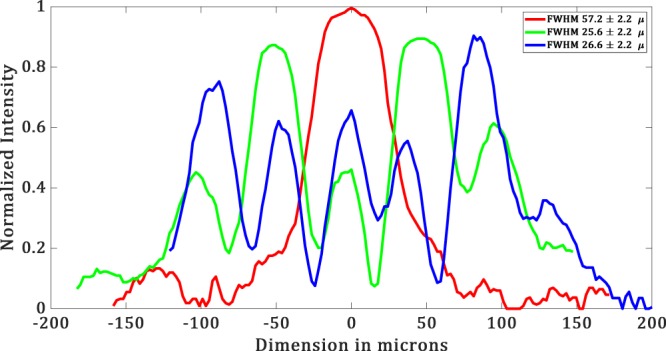
Intensity profile plots of the experimental images in Figure 15(a–c).

The experimental results are shown in Figure 15(a–c) and the corresponding simulated results are shown in Figure 15(d–f). From the intensity profile plots (see Figure 16 and 17) of the images in Figure 15, we see that the simulated results are well in agreement with the experimental results. For simulated hotspot of size 23 ± 1 *μm*, we get ~25.6 ± 2.2 *μm* in the experiment (see Figure 15(b)). In this case, we have a *FOV* of 66 *μm* in the experiment versus 71 *μm* in simulation. The intensity ratio between the maximum of the hotspot to that of the side lobes is equal to 0.52 in the experiment whereas in the simulation it is about 0.91. This deviation is probably due to the non-uniformity in the illumination. In Figure 15(c), we obtain a hotspot of size ~26.6 ± 0.2 *μm* versus 25 ± 1 *μm* for the simulated hotspot.

In this case, the simulated hotspot shows an increased *FOV* of 150 *μm* between the central hotspot and the main bright sidelobe. There are minor sidelobes whose intensities are lower than the central hotspot. In the experiment, we get considerably similar results with an increased *FOV* of 154 *μm*. However, the intensity of the minor sidelobes in the experiment is not similar to that of the simulation. Still, they are almost equal or less than the central hotspot. The reason for this discrepancy can be attributed to the non-uniformity in illumination. The intensity ratio of the central spot to the main side lobes, in this case, is equal to 0.72 versus 0.87 in the experiment. Hence, we get an increased *FOV* with an increased Intensity ratio.

## Conclusion

We have presented a novel technique for the generation and manipulation of superoscillatory hotspots. Our work brings the concept of CTF shaping to the superoscillatory research regime, which was not yet investigated before. The uniqueness of our method is that, we don’t use complex mathematics for the generation of a hotspot, rather our method produces hotspots of different sizes based on the tuning of the Fourier filter mask that we introduce in the digital optimization.

In our work, we also showed the concept of increasing the FOV with a considerable intensity ratio with the hotspot to sidelobe and less loss in resolution of the generated hotspot. We hope that our technique will become an important tool in a range of applications and initiate other scientific researches using this tool.

## Supplementary information

Supplementary information.
