# Author Correction: Generation and Manipulation of Superoscillatory Hotspots Using Virtual Fourier Filtering and CTF Shaping

**DOI:** 10.1038/s41598-020-73511-4

**Published:** 2020-10-01

**Authors:** Abhijit Sanjeev, Nadav Shabairou, Arrad Attar, Daniel Scherbaum, Yuval Kapellner, Moshe Sinvani, Zeev Zalevsky

**Affiliations:** 1 Faculty of Engineering and the Institute for Nanotechnology and Advanced Materials, Bar-Ilan University, 5290002 Ramat-Gan, Israel; 2 EKB Technologies Ltd, 59513 Bat-Yam, Israel; 3 Erlangen Graduate School in Advanced Optical Technologies (SAOT), Paul Gordan-Strasse 6, 91052 Erlangen, Germany

Correction to: *Scientific Reports* 10.1038/s41598-020-61674-z, published online 16 March 2020

The original version of this Article contained a typographical error in the spelling of the author Daniel Scherbaum, which was incorrectly given as Daniel Scheberbaum. This has now been corrected in the PDF and HTML versions of the Article, and in the accompanying Supplementary Information file.

