# Supplementary material for: Generation and Manipulation of Superoscillatory Hotspots Using Virtual Fourier Filtering and CTF Shaping
Source: Sci Rep. 2020 Mar 16;10:4755. doi: 10.1038/s41598-020-61674-z (PMC7075986; doi:10.1038/s41598-020-61674-z)
Supplement: Supplementary file 1 — Supplementary information. [file 41598_2020_61674_MOESM1_ESM.pdf]

Generation and Manipulation of Superoscillatory Hotspots Using Virtual Fourier Filtering and CTF Shaping

Abhijit Sanjeev[1,2*], Nadav Shabairou [1], Arrad Attar [1], Daniel Scheberbaum[3], Yuval Kapellner[2], Moshe Sinvani[1] and Zeev Zalevsky[1]

[1]Faculty of Engineering and the Institute for Nanotechnology and Advanced Materials, Bar-Ilan University, Ramat-Gan 5290002, Israel
[2]EKB Technologies Ltd, Bat-Yam 59513, Israel
[3] Erlangen Graduate School in Advanced Optical Technologies (SAOT), Paul Gordan-Strasse 6, 91052, Erlangen
**Correspondence and requests for materials should be addressed to A.S. (abhijitsanjeevk@ gmail.com)*

Supplementary Material

Simulation Results

Table S1 and S2 consolidate the simulation results obtained for $\lambda = 460\ nm\ and\ 632\ nm.$ The simulation was performed for $NA = 1$. In both cases the value of r_1, r_2 and r_3 fall within the range we

$MASK$ #	$r_1\ (nm)$	$r_2(nm)$	$r_3(nm)$	$Hotspot\ Size\ (nm)$	$FOV\ (nm)$
1	73.15	279.3	385.7	166.1 ± 6.65	472.1 ± 6.65
2	119.7	279.3	385.7	139.5 ± 6.65	432.1 ± 6.65
3	133	279.3	385.7	$112.9\ \pm 6.65$	418.9 ± 6.65
4	172.9	279.3	385.7	46.4 ± 6.65	352.3 ± 6.65
5	66.5	618.45	711.5	$126.2\ \pm 6.65$	964.1 ± 6.65

mentioned

Table S1: Simulation Results for $\lambda = 460\ nm, NA = 1$.

$MASK$ #	$r_1\ (nm)$	$r_2(nm)$	$r_3(nm)$	$Hotspot\ Size\ (nm)$	$FOV\ (nm)$
1	73.1500	379.0500	518.7	$192.7\ \pm 6.65$	$631.9\ \pm 6.65$
2	119.7	379.0500	518.7	$179.7\ \pm 6.65$	$592.1\ \pm 6.65$
3	133	379.0500	518.7	$166.5\ \pm 6.65$	$578.7\ \pm 6.65$
4	172.9	379.0500	518.7	$113.2\ \pm 6.65$	$511.9\ \pm 6.65$
5	66.5	711.5	844.5	$206.3\ \pm 6.65$	$1336\ \pm 6.65$

Table S2: Simulation Results for $\lambda = 632\ nm, NA = 1$.

in the main manuscript. In Fig. S3 we show the simulation results for $\lambda = 780\ nm, NA = 0.5$ and the pixel size of psf plane is 30 nm

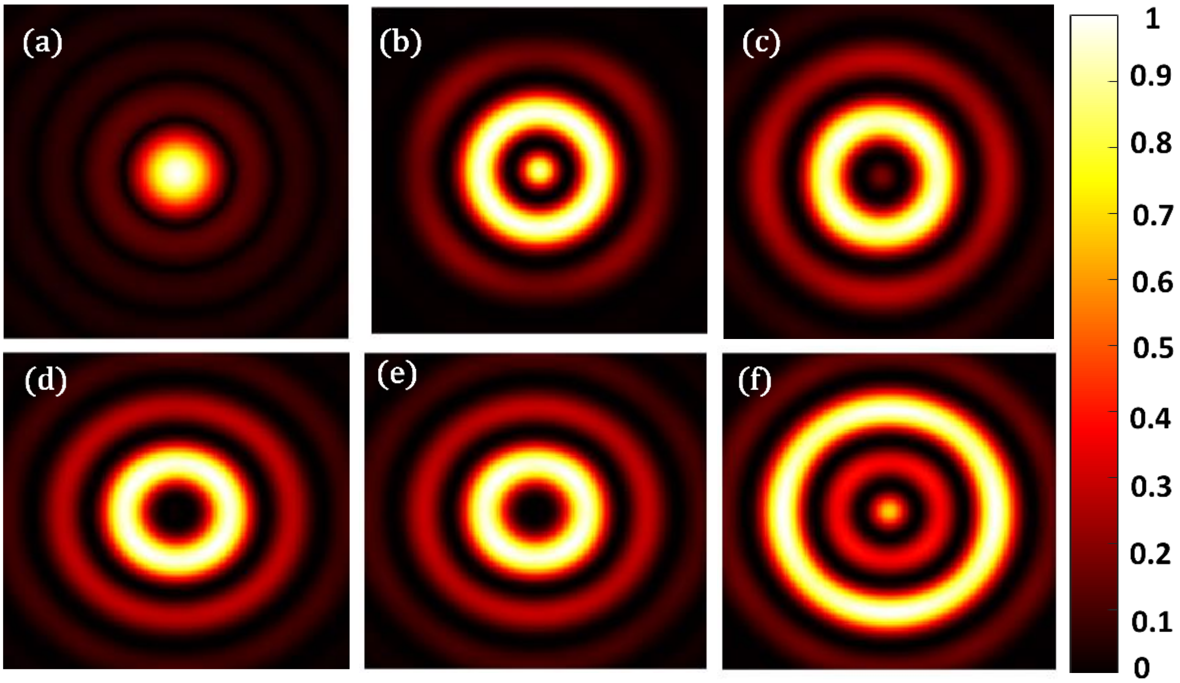

Figure S3. Simulation results for $\lambda = 780\,nm, NA = 0.5$ (a) Diffraction limited spot of $FWHM$ size $951.6\,nm$. Superoscillatory hotspot of sizes (b $510 \pm 30\,nm$ (c) $390\ \pm 30\,nm$ (d) $210 \pm 30\,nm$ (e) $150\ \pm 30\,nm$. (f) Superoscillatory hotspot of size $510 \pm 30\,nm$ nm with an extended FOV of $3270\ \pm 30\,nm$

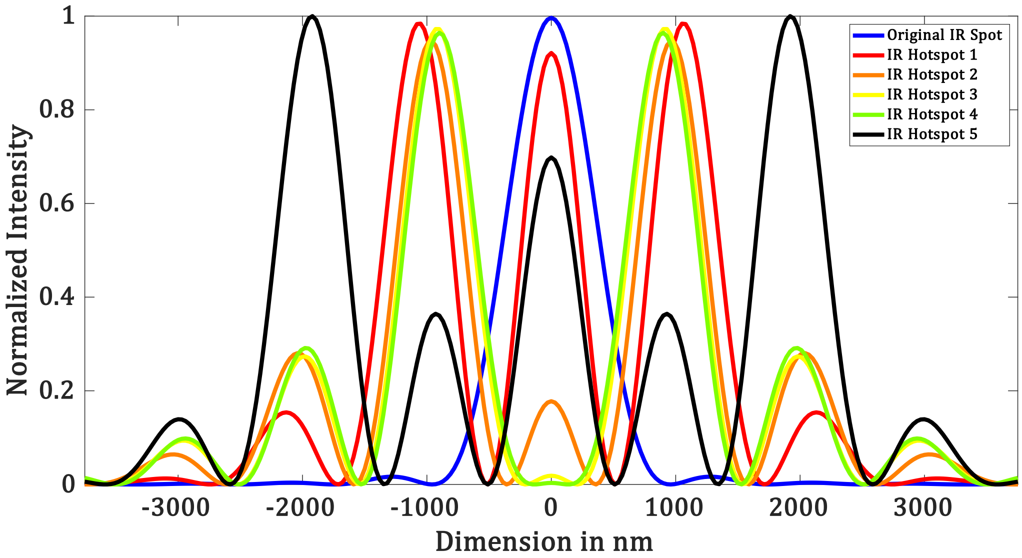

Figure S4. Intensity profile plots of the simulated results in Fig. S3. Blue plot refers to the intensity profile plot of Fig S3 (a). red plot refers to the intensity profile plot of Fig S3 (b), black plot refers to the intensity profile plot of Fig S3 (f), orange plot refers to the intensity profile plot of Fig S3 (c), yellow plot refers to the intensity profile plot of Fig S3 (d) and green plot refers to the intensity profile plot of Fig S3 (e).

Table S5 consolidates the simulation results obtained in Fig. S3.

$MASK$ #	$r_1\ (nm)$	$r_2(nm)$	$r_3(nm)$	$Hotspot\ Size\ (nm)$	$FOV\ (nm)$
1	270	960	1290	$510\ \pm 30$	1590 ± 30
2	480	960	1290	$390\ \pm 30$	$1350\ \pm 30$
3	540	960	1290	210 ± 30	1230 ± 30
4	570	960	1290	$150\ \pm 30$	1230 ± 30
5	420	1740	2100	510 ± 30	$3270\ \pm 30$

Table S5: Simulation Results for $\lambda = 780\ nm, NA = 0.5$.

In order to generalize our criteria for the selection of the mask parameters, we did try to simulate the same for a few other wavelengths as well as different NA systems. All of them tend to follow the same trend in the selection of r_1, r_2 and r_3 in yielding a hotspot. Hence, we come to the conclusion that in order to get a hotspot value of r_1 should be such that $\frac{r_1}{r_2} > 0.25$ and $r_1 < FWHM$ of the diffraction-limited spot.

In Fig. S6 we show additional simulation results on $\lambda = 532\ nm, NA = 1$. Here we show that indeed it is possible to extend the FOV further. It is possible to set the value of r_2 and r_3 based on the third minor lobes in the diffraction-limited amplitude pattern. It will be useful in expanding further the FOV, with a hotspot that is still less than the diffraction limit. However, in such cases, the intensity of such hotspots will be very less and most of the energy will be concentrated in the side lobes. So the payment you give is in the intensity. So we have to always look for a fine-tuning between the hotspot size, peak intensity ratio and the FOV. An additional point to be noted is that the choice of the third minor lobes doesn't guarantee the creation of hotspot. Since the G.S Algorithm, we are using starts with a random phase function, it is hard for it to converge to a hotspot. In order to solve this problem, we used the phase mask that we obtained in the creation of the hotspot using the first minor side lobes as a seed function to the algorithm. Fig. S7 shows the intensity profile of Fig. S6

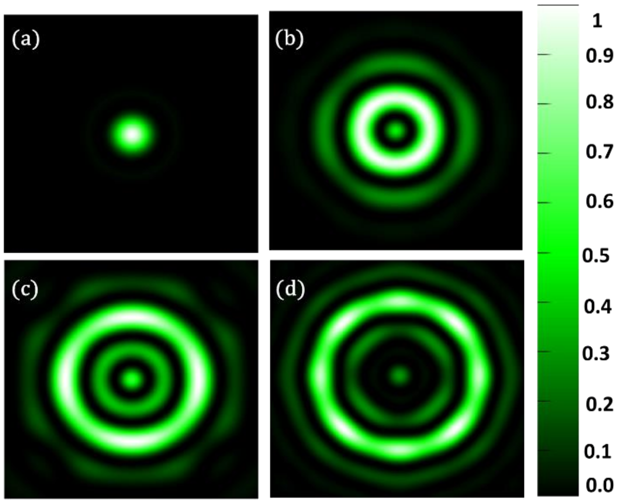

Figure S6. (a) **.** Simulation results for $\lambda = 532\ nm, NA = 1$ (a) Diffraction-limited spot of size $324.5 \pm 6\ nm$. Superoscillatory hotspot of sizes (b) 153.1 $\pm$ 6.65 nm (c) 166.4 ± 6.65 nm with an extended FOV of $1123.7 \pm 6.65\ nm$. (d) $166.4 \pm 6.65\ nm$ with further extended FOV of $1602.9\ \pm\ 6.65\ nm$.

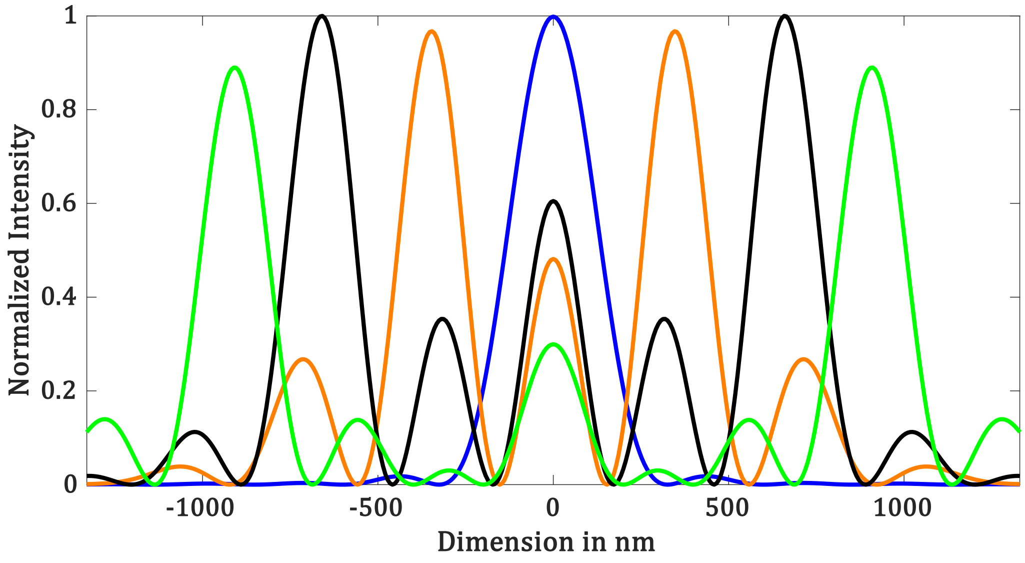

Figure S7. Intensity profile plots of the simulated results in Fig. S3. Blue plot refers to the intensity profile plot of Fig S6 (a).the orange plot refers to the intensity profile plot of Fig S6 (b), black plot refers to the intensity profile plot of Fig S6 (d) and green plot refers to the intensity profile plot of Fig S6 (e).

Based on our analysis, we did not obtain a hotspot when we chose arbitrary values for r_2 and r_3. The reason is because of the nature of the optimization problem finding hard to converge.
